# Derivation of normative data for the COPD assessment test (CAT)

**DOI:** 10.1186/1465-9921-15-68

**Published:** 2014-06-23

**Authors:** Lancelot M Pinto, Nisha Gupta, Wan Tan, Pei Z Li, Andrea Benedetti, Paul W Jones, Jean Bourbeau

**Affiliations:** 1 Respiratory Epidemiology and Clinical Research Unit, McGill University Health Centre, Montréal, Québec, Canada; 2 ICapture, University of British Columbia, Vancouver, British Columbia, Canada; 3 Division of Clinical Science, St George’s, University of London, London, UK; 4 Department of Medicine, McGill University, Montréal, Canada; 5 Department of Epidemiology, Biostatistics & Occupational Health, McGill University, Montréal, Canada

**Keywords:** COPD assessment test, Health-related quality of life, Normative data, Questionnaire

## Abstract

**Background:**

The tradition classification of the severity of COPD, based on spirometry, fails to encompass the heterogeneity of the disease. The COPD assessment test (CAT), a multi-dimensional, patient-filled questionnaire, assesses the overall health status of patients, and is recommended as part of the assessment of individuals with COPD. However, information regarding the range of values for the test in a non-COPD population (normative values) is limited, and consequently, knowledge regarding the optimal cut-off, and the minimum clinically important difference (MCID) for the test remain largely empirical.

**Methods:**

CanCOLD is a population-based multi-center cohort study conducted across Canada, the methodology of which is based on the international BOLD initiative. The study includes subjects with COPD, at-risk individuals who smoke, and healthy control subjects. CAT questionnaires were administered at baseline to all subjects. Among non-COPD subjects, normative values for the CAT questionnaire, and psychometric properties of the test were characterized. Predictors of high CAT scores were identified using multivariable logistic regression.

**Results:**

Of the 525 non-COPD subjects enrolled, 500 were included in the analysis. Mean FEV_1_/FVC ratio among the 500 included subjects was 0.77 (SD 0.49); the mean predicted FEV_1_ was 99.38% (SD 16.88%). The overall mean CAT score was 6 (SD 5.09); scores were higher among females (6.43, SD 5.59), and subjects over 80 years of age (mean 7.58, SD 6.82). Cronbach alpha for the CAT was 0.79, suggesting a high internal consistency for the test. A score of 16 was the 95th percentile for the population, and 27 subjects (5.4%) were found to have a CAT score > =16. Current smoking (aOR 3.41, 95% CI 1.05, 11.02), subject-reported physician-diagnosed asthma (aOR 7.59, 95% CI 2.71, 21.25) and musculoskeletal disease (aOR 4.09, 95% CI 1.72, 9.71) were found to be significantly associated with a score ≥16.

**Conclusions:**

The characterization of CAT scores in the general population will be useful for norm-based comparisons. Longitudinal follow-up of these subjects will help in the optimization of cut-offs for the test.

## Background

Chronic Obstructive Pulmonary Disease (COPD) is the 4th leading cause of mortality worldwide, and causes significant morbidity [1,2]. Though spirometry is required for the diagnosis of COPD, it is not a substitute for measuring patient perspective with respect to symptoms, function or overall health condition. More recently, the GOLD (Global Initiative for Chronic Obstructive Lung Disease) strategy document has acknowledged the increasing evidence that FEV_1_ alone is inadequate for describing COPD status [3]. The new recommended approach represents a move towards individualized treatment for COPD patients, matching therapy more closely to a multi-dimensional assessment of specific patient attributes such as symptoms, spirometric classification and evaluation of the risk of future adverse events, particularly exacerbations. GOLD recommends the COPD Assessment Test (CAT) or mMRC scores to differentiate between patients experiencing a low or high burden of symptoms.

The development of the CAT questionnaire and selection of the items was done based on the testimonies of patients about COPD and the effect that it has upon them [4]. The final CAT consists of eight items, each formatted as a six-point differential scale, making the tool easy to administer [5]. The psychometric properties of the questionnaire have been studied and validated in the patient population in the clinical setting, and the test appears to have a good construct and discriminant validity [5-8], and is responsive to changes in health status of patients with COPD [9-11]. A study from our group, which included both COPD and non-COPD subjects selected from the population, and from one of the sites of the cohort included in the present study, suggested that the overall CAT score had a statistically significant association with a diagnosis of COPD [12].

Although the CAT questionnaire was developed for use among patients with COPD, the interpretation of measures of COPD health status may represent a considerable challenge with respect to the magnitude and gravity of the scale of responses. Norm-based comparisons, derived from the general, non-COPD population, are a strategy that can help resolving these challenges. They can also help in testing hypotheses for clinical trials that are targeted at improving patient-centered outcomes, knowing the clinical relevance of a score to adequately power such trials. The CAT questionnaire has been tested in general population in the Middle East, as part of the BREATHE study [8], although the participants were poorly characterized in terms of physiological measurements to confirm the presence or absence of COPD. It has also been tested among healthy industrial workers in Japan [13]. Normative values for the test in a Western setting are not known.

The present study was performed to gain population norms for CAT in a well-characterized, population-based sample. The primary objective of the present study was to derive the normative values for the CAT questionnaire for the adult general population (≥40 years of age), and the norms by age and sex. The secondary objectives included: a) to identify subjects who have a CAT score that was equal to, or greater than the 95th centile for this population, and to identify predictors, using an adjusted multivariable analysis, that were different in these subjects when compared to individuals with scores below the 95th centile; b) to test the psychometric properties of CAT in the general population, including internal consistency and validity.

## Methods

### Study population

The Canadian Cohort Obstructive Lung Disease (CanCOLD) study is a prospective longitudinal cohort study that is designed to track 1500 subjects across Canada (recruited using a random sampling frame from the populations of 9 urban/suburban areas) [14]. The study is based on the international BOLD initiative [15]. The cohort comprises two COPD balanced subsets (GOLD ≥ 2 and GOLD 1) and two age- and sex- matched non-COPD peers (ever-smoker at risk and healthy controls, i.e. never smokers with post-BD FEV1/FVC > 0.70). Assessments are made at baseline, 18 months, 3 years and beyond, and include for non-COPD subjects the CAT questionnaire the SF-36 questionnaire, complete pulmonary function and cardio pulmonary exercise assessments, CT scans of the chest and blood tests. The details of the sampling strategy and assessments have been described in the published protocol [14]. For the present study, we used the CAT, filled at baseline, by the subjects recruited in the non-COPD subsets (ever-smoker at risk, healthy non-smoker controls) of the CanCOLD study. The CanCOLD study was approved by the REB at the McGill University Health Centre (MUHC) - Study # 09-025-BMC.

### CAT questionnaire

**Figure 1:**
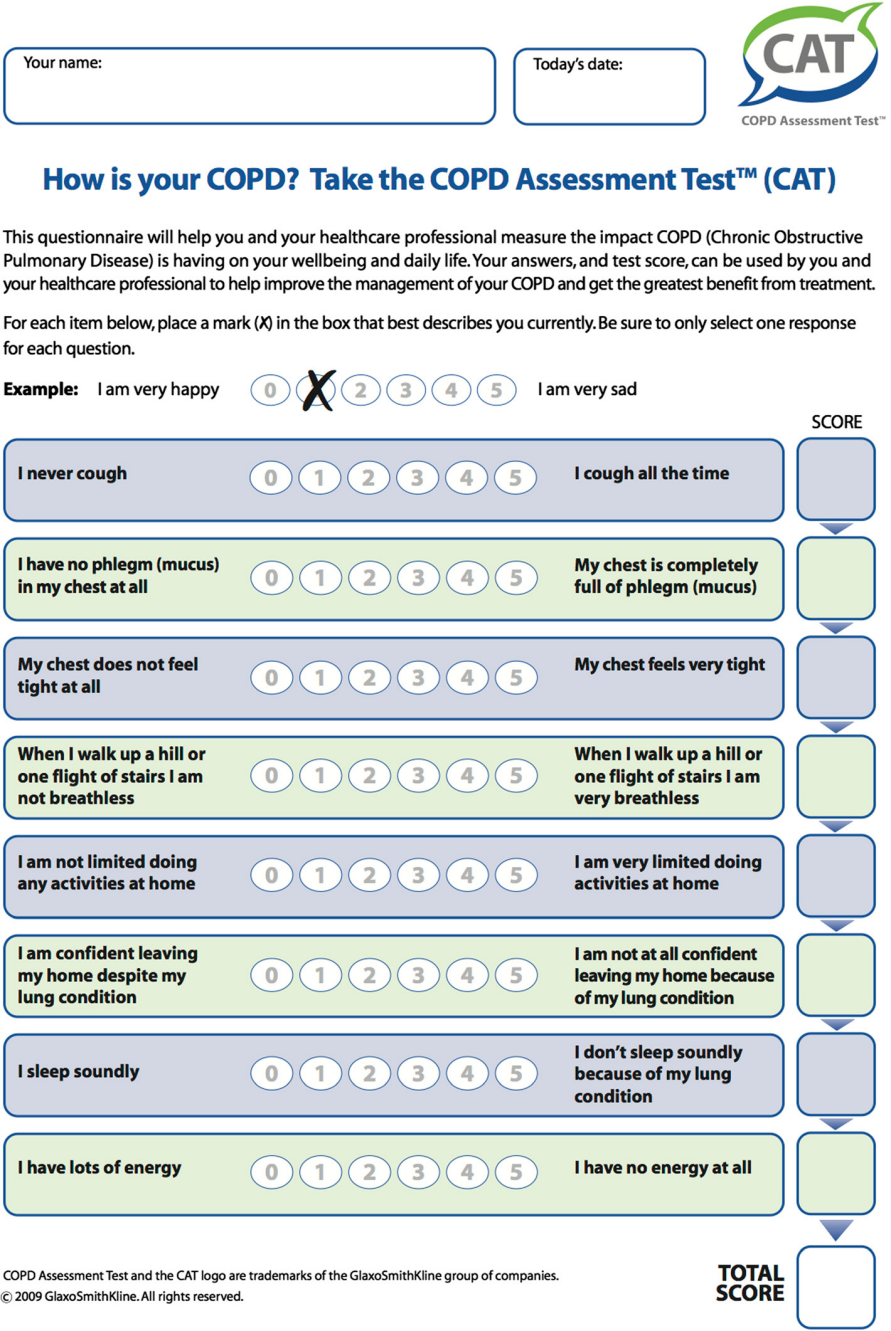
COPD Assessment Test Questionnaire.

The CAT (Figure 1) has 8 questions (items) and includes constituent items that assess cough, production of phlegm, chest tightness, breathlessness, activity limitation, confidence, sleep and energy. The minimum score possible (floor) for each item is zero, and the maximum (ceiling) is five. Thus, the overall score can have a value ranging from 0 – 40.

### Normative values for the CAT questionnaire

Normative values for the CAT questionnaire were described using the mean, standard deviation, range (minimum and maximum), distribution of scores among males and females by percentiles, the percentage of responders at the lowest score possible (floor) and the highest score possible (ceiling) for the overall score, categorized by age groups. Participants were divided into five age categories, 40–49 years, 50–59 years, 60–69 years, 70–79 years and 80 years and above.

### Identification of predictors of high CAT scores

From the descriptive statistics, we dichotomized subjects into those with CAT scores equal to and above the 95th centile in the study population (defined as a high CAT score), and those with scores below the 95th centile. We assessed the association of a high CAT score with variables defined *a priori*. These included sex, age, being a current smoker, forced expiratory volume in the first second (FEV_1_) as a percentage of the volume predicted by the NHANES equation for the subject, and presence of comorbidities including physician-diagnosed asthma, cardiovascular disease and musculoskeletal disease. Variables found to be significantly associated with a high CAT score were then analyzed using a multivariable logistic regression model to identify which of the identified variables were predictors of a high CAT score. We also performed the above analysis using the suggested cut-off of 10 for the CAT score being abnormal.

### Psychometric properties of CAT

i) Face validity

We analyzed the overall CAT scores among smokers and non-smokers by sex to assess face validity.

ii) Internal consistency

The internal consistency of the eight items in the CAT was assessed using Cronbach alpha, which is a coefficient of internal consistency. It is a function of the correlation of an item on the questionnaire with the overall test score (item-test correlation) and with the other items on the questionnaire (item-rest correlation). Values are graded as excellent (≥0.9), good (0.8 ≤ α < 0.9), acceptable (0.7 ≤ α < 0.8), questionable (0.6 ≤ α < 0.7), poor (0.5 ≤ α < 0.6) and unacceptable (<0.5) [16].

iii) External validity

Canadian normative data for the SF-36 health questionnaire have been published [17] and were used as the reference standard for assessing the validity of the CAT. The Short Form 36 (SF-36) is a generic health related quality of life (HRQOL) questionnaire that includes eight multi-item scales. These include physical functioning (10 items), physical role limitations (4 items), emotional role limitations (3 items), bodily pain (2 items), social functioning (2 items), mental health (5 items), vitality (4 items), general health perceptions (5 items), and health transition (1 item) [18].

The domains of the SF-36 have also been aggregated to create composite measures of physical and mental health. The physical functioning, role physical, bodily pain, and general health scales are used to derive and aggregate physical health summary measure, and the role emotional, social functioning, mental health and vitality scales are aggregated to derive a mental health summary measure [19]. We analyzed the correlation of the overall CAT score, with the aggregate physical and mental summary health measure of the SF-36 using Pearson’s correlation coefficient as a measure of the external validity of the test as a measure of the health status. All data were analyzed using STATA version 11.0 (Statacorp, TX, USA).

## Results

### Normative values, and psychometric properties of the CAT

525 non-COPD subjects have been recruited in the study. CAT questionnaires were missing for 25 subjects (4.8%), so 500 subjects were included in the analysis. There was no significant difference found in the demographic characteristics included and excluded subjects. As compared to the Canadian general population, individuals in the 40–49 year-old group were under-represented in this cohort, while individuals in the older age groups appear to be over-represented. Almost two third of these individuals were retired from work and less than 10% were current smokers. The SF-36 scores for the included subjects were similar to those reported as normative data for the Canadian population [17]. The mean FEV_1_/FVC ratio was 0.77 (SD 0.49) with a mean predicted FEV_1_ of 99.38% (SD 16.88%). The demographic characteristics of the subjects are summarized in Table 1.

**Table 1:** **Demographic and clinical characteristics of the non**-**COPD participants**

**Age group, years**	
40-49	26 (5.2)
50-59	92 (18.4)
60-69	184 (36.8)
70-79	160 (32)
> = 80	38 (7.6)
**Gender**	
Male (M)	240 (48)
Female (F)	260 (52)
**Occupation**	
Retired	307 (61.4)
Full-time	105 (21)
Part-time	57 (11.4)
Disability	14 (2.8)
Unemployed and homemaker	17 (3.4)
**Smoking status**	
Never smoker	246 (49.2)
Ex-smoker	221 (44.2)
Current smoker	33 (6.6)
**Comorbidities**	
Hypertension	98 (19.6)
Diabetes	54 (10.8)
Asthma	31 (6.2)
Cardiac disease	78 (15.6)
Musculoskeletal disease	181 (36.2)
**SF-36 scores (Mean, SD)**	
Physical component scale, M and F	50.95 (8.47) and 51.6 (8.73)
Mental component scale, M and F	50.65 (8.62) and 50.95 (9.82)
**Spirometric measures (Mean, SD)**	
FEV1/FVC ratio	77.14 (4.86)
FEV1 %predicted	99.38 (16.88)

SF-36: Short-form-36 questionnaire.

FEV_1_: forced expiratory volume, 1 second.

FVC: forced vital capacity.

**Figure 2:**
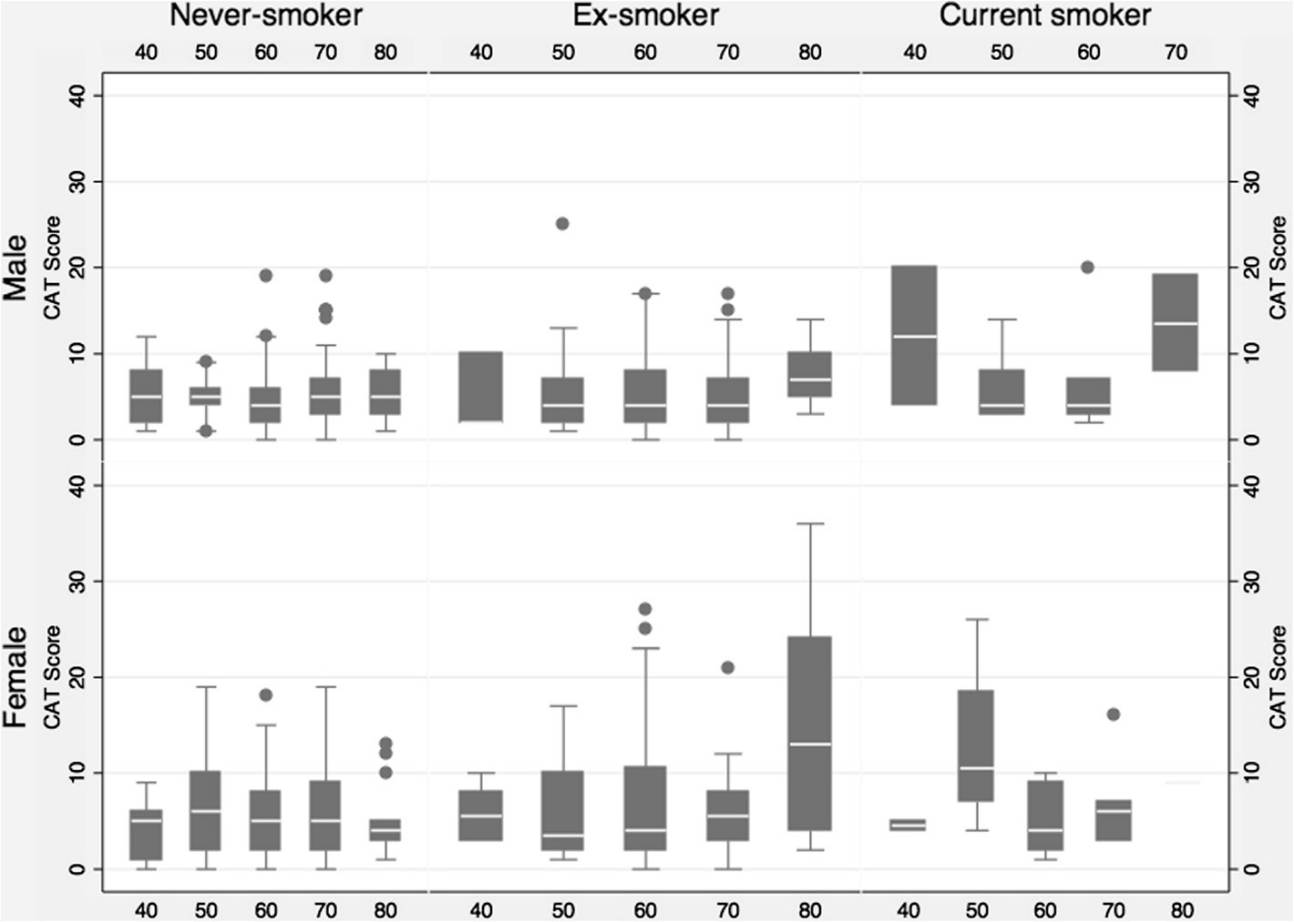
**Normative values, and distribution (by percentiles) for the overall CAT score by age group and sex.** CAT: COPD assessment test (scores range from 0 – 40). Boxes represent the inter-quartile range with the white line within the box representing the median. Whiskers represent extreme values within 1.5 IQR of the lower and upper quartiles, respectively. Values beyond this (outliers) are represented by discrete points in the graph.

The Cronbach alpha for the CAT was 0.79, suggesting a high internal consistency in this population. The overall correlation coefficient for the test with the physical component of the SF-36 was -0.48, and with the mental component was -0.38. The normative values, and distribution (by percentiles) for the overall CAT score by age group and sex is summarized in Table 2. The overall mean score was 6 (SD 5.09), and it was higher in females (6.43, SD 5.59) as compared to males (5.53, SD 4.45). The overall inter-quartile range was 2–8 (3–8 for males 2–9 for females). CAT scores were similar across the age strata from 40–79 (mean score range for each strata 5.54-6.37), but were higher for the group of subjects over 80 years of age (mean 7.58, SD 6.82) (Figure 2). Overall values ranged from 0 to 36.

**Table 2:** **Normative values for the CAT score in the non**-**COPD participants**

	**Males**						**Females**						**Overall**
**Age group**	**40-49 years**	**50-59 years**	**60-69 years**	**70-79 years**	**80 years and above**	**All ages**	**40-49 years**	**50-59 years**	**60-69 years**	**70-79 years**	**80 years and above**	**All ages**	**All ages**
Number of subjects (%)	12 (2)	43 (9)	85 (17)	82 (16)	18 (4)	240 (48)	14 (3)	49 (10)	99 (20)	78 (16)	20 (4)	260 (52)	500
Minimum score	1	1	0	0	1	0	0	0	0	0	1	0	0
5th percentile	1	1	0	0	1	0	0	1	0	1	1.5	0.5	0
10th percentile	2	2	1	1	3	1	1	2	1	1	2	1	1
25th percentile	2	3	2	2	4	3	3	2	2	3	3	2	2
50th percentile	5	5	4	5	6.5	4	5	6	5	5	4	5	5
75th percentile	9	6	7	8	10	8	8	10	8	9	11	9	8
90th percentile	12	10	10	14	12	12	9	17	12	13	21	13	12
95th percentile	20	13	12	15	14	15	10	19	21	16	30	18	16
Maximum score	20	25	20	19	14	25	10	26	27	21	36	36	36
Mean score	6.58	5.47	4.96	5.72	6.72	5.53	5.00	7.16	6.04	6.23	8.35	6.43	6
Standard Deviation, SD	5.48	4.39	4.18	4.78	3.49	4.45	2.96	5.76	5.58	4.69	8.85	5.59	5.09
Percentage of subjects at floor^a^	0	0	8.2	7.3	5.5	5.4	7	2	8	3.8	5	5	5.2
Percentage of subjects at ceiling^b^	0	0	0	0	0	0	0	0	0	0	0	0	0

^a^Floor represents the lowest score possible (zero for the overall score).

^b^Ceiling represents the highest score possible (40 for the overall score).

CAT: COPD assessment test.

### Identification of predictors of high CAT scores

A score of 16 was found to be at the 95th percentile for the study population. 27 subjects (5.4%) were found to have a high CAT score (defined as > =16). The group comprised 8 of the 246 subjects who were non-smokers (3.25%), 14 of the 221 subjects who were ex-smokers (6.33%), and 5 of the 33 subjects who were current smokers (15.15%). Current smoking status had a significant effect (OR 3.61, 95% CI 1.27,10.25)., The presence of physician-diagnosed asthma reported by the subject (OR 8.24, 95% CI 3.26, 20.8), and subject reporting to have musculoskeletal disease (OR 3.8, 95% CI 1.67, 8.66) were statistically significant in the univariate analysis. Lower CAT scores imply a better health status, and consistent with what was expected, there was an inverse correlation between CAT scores and predicted FEV_1_ (OR 0.97, 95% CI 0.95, 0.99). In the multivariable logistic regression, current smoking (aOR 3.41, 95% CI 1.05, 11.02), physician-diagnosed asthma (aOR 7.59, 95% CI 2.71, 21.25) and subject –reported musculoskeletal disease (aOR 4.09, 95% CI 1.72, 9.71) were found to be significantly associated with a high CAT score.

When classified based on the suggested cut-off score of 10, 96 subjects (19.2%) were found to have an abnormal CAT score. In the adjusted analysis, an increase in the FEV_1_ percentage predicted, existing cardiovascular disease and having physician-diagnosed asthma reported by the subject were the only factors found to be significantly associated with a high CAT score. Musculoskeletal disease was not a significant effect in this analysis. Smoking was not found to be associated with a high CAT score (OR 1.64, 95% CI 0.74, 3.65). The results of the analyses are summarized in Table 3. 254 subjects did not have any of the above-mentioned comorbidities. The mean CAT score in this subset of subjects was 5.11 (SD 4.1).

**Table 3:** **Predictors of a high CAT score**^**a**^

**Variable**	**OR (95% CI) for a high CAT score (≥95th percentile, score of 16) in non-COPD participants**	**Adjusted OR (95% CI) for a high CAT score**^**a**^
Male sex	0.44 (0.19,1.02)	
Increase in age	1 (0.96, 1.04)	
Current smoker	3.61 (1.27,10.25)	3.41 (1.05, 11.02)
FEV_1_ percentage predicted	0.97 (0.95-0.99)	0.99 (0.96, 1.01)
Patient reported having asthma	8.24 (3.26,20.8)	7.59 (2.71, 21.25)
Patient reported having cardiovascular disease	1.59 (0.62,4.08)	
Patient reported having musculoskeletal disease	3.8 (1.67,8.66)	4.09 (1.72, 9.71)
**Variable**	**OR (95% CI) for a high CAT score (defined here as CAT score ≥ 10) in non-COPD participants**	**Adjusted OR (95% CI) for a high CAT score**^**a**^
Male sex	0.62 (0.39,0.98)	0.66 (0.4, 1.08)
Increase in age	1 (0.98, 1.03)	
Current smoker	1.64 (0.74, 3.65)	
FEV_1_ percentage predicted	0.97 (0.96-0.99)	0.98 (0.96, 0.99)
Patient reported having asthma	3.36 (1.58,7.92)	2.3 (1.02, 5.19)
Patient reported having cardiovascular disease	2.51 (1.47,4.3)	2.35 (1.32, 4.18)
Patient reported having musculoskeletal disease	1.56 (1, 2.46)	1.46 (0.9, 2.37)

*CAT* COPD assessment test; *OR* odds ratio.

^a^Two definitions of a high CAT score were used, the first was ≥95th percentile, and the second was ≥ 10, which is considered as the conventional cut-off.

## Discussion

This is one of the first studies that describe the normative values of the CAT questionnaire in a population-based sample of healthy subjects and well characterized in terms of physiological measurements to confirm the presence or absence of COPD. This will serve as a valuable benchmark for norm-based future comparisons.

The overall mean score for the CAT (6, SD 5.09) in this population was within the range of values found for the Arabic, Japanese, and Turkish versions tested in large non-COPD populations (mean scores of 5.4, 6.3 and 8.07 respectively) [8,13]. Women and older individuals (over 80 years of age in the present study) had higher CAT scores, consistent with the results from the BREATHE study [8]. The 95th centile for overall CAT score for this study (score of 16) was lower than that found for the Arabic and Turkish versions (21 and 28, respectively) [8]. The relationship observed between CAT score and smoking status, suggests, that even in an unselected population, the CAT might be a useful screening tool for the assessment of respiratory health. In the adjusted analysis, a prior diagnosis of asthma by a physician appeared to have the strongest correlation with a high CAT score, followed by the association with a current smoking status, and these results possibly reflect the specificity of the questionnaire for respiratory health. The statistically significant association of a high score with musculoskeletal disease suggests that CAT scores may be slightly influenced by limitation of activity due to other conditions, although this effect disappeared when the participants were characterized into higher and lower CAT scores. The loss of the association between high CAT scores and smoking status, when a cut-off of 10 was used, suggests that a lower cut-off may result in a greater proportion of abnormal scores being a consequence of non-respiratory conditions, and this may be reflected by the presence of an association with patient-reported cardiovascular disease. However, further studies with an attempt to anchor the score to physiological, functional and chest radiological imaging parameters in the normal population would be needed to derive the optimal cut-off for the score when used in this way.

Results from the testing of the psychometric properties of the questionnaire were consistent with the available literature, suggesting the CAT has good internal and external validity. The lack of a strong correlation of the CAT score with both, the physical and the mental component of the SF-36, suggests that the CAT may be more suited to assessing respiratory, and not overall quality of life.

Our study has several strengths. The population sampling strategy based on the international study BOLD, the low missing data, and the consistency of the SF-36 scores obtained from the subjects in this study, suggest that selection biases may have been minimized. The study by Nishimura et al. [13] described normative values for the CAT score in healthy industrial workers from Japan, thereby potentially being biased due to the “healthy worker effect” [20]. The study also did not use post-bronchodilator spirometric values for the diagnosis of COPD, thereby potentially misclassifying individuals with, and without COPD. Our study was a population-based study, and used post-bronchodilator values, as recommended by the guidelines [3].

When compared to the overall Canadian population, individuals in the age group of 40–49 years appear to have been under-represented. Also, a significant proportion of the individuals in our study were retired from work because of the age group selected, and the proportion of individuals with comorbidities also reflects the ageing population, unlike the study by Nishimura et al. [13] which comprised a working population. Finally, the subjects studied in our study were primarily Caucasian, with an underrepresentation of other ethnicities. These could represent potential selection biases, and are limitations of the study.

## Conclusion

In conclusion, our study provides normative values for the CAT questionnaire from well characterized individuals in a general population, to be used for norm-based comparisons. We have shown that current smoking status, physician-diagnosed asthma and musculoskeletal disease are predictors of a higher CAT score. Consistent with available literature, the CAT had a high validity not only in COPD but also in this general population. Follow-up in the longitudinal CanCOLD study will be useful in assessing the optimal cut-off for score, and the association of changes in respiratory physiological and morphometric (CT Scan imaging) measurements with changes in health status as measured by the CAT questionnaire.

## Competing interests

The authors declare that they have no competing interests.

## Authors’ contributions

LP - planned and created the protocol, analyzed the data, wrote and reviewed the article. NG - reviewed the article WT - reviewed the article and critiqued the methodology PL - assessed the quality of the statistical analysis and reviewed the article AB - assessed the quality of the statistical analysis and reviewed the article PJ - reviewed the article and critiqued the methodology JB-planned and created the protocol, wrote and reviewed the article. All authors read and approved the final manuscript.
